# COVID-19 and Its Impact on Back Pain in the Eastern Province of Saudi Arabia

**DOI:** 10.7759/cureus.57475

**Published:** 2024-04-02

**Authors:** Bushra M Alsayari, Shahad M Alshehri, Abdullatif Y Almulhim, Leena M Alzakry, Abdullatif A Alzuraiq, Fahad H Binshalhoub, Hanin M Banjer, Lama Mohammed A Alkhediwi, Kholood M Rasdwi, Abdul Sattar Khan

**Affiliations:** 1 Medicine and Surgery, King Faisal University, Al-Hasa, SAU; 2 College of Medicine, King Faisal University, Al-Hasa, SAU; 3 Medicine, Imam Muhammad Ibn Saud Islamic University, Riyadh, SAU; 4 Medicine and Surgery, Imam Muhammad Ibn Saud Islamic University, Riyadh, SAU; 5 College of Medicine, King Abdulaziz University, Jeddah, SAU; 6 College of Medicine, Al-Baha University, Al-Baha, SAU; 7 Family Medicine, King Faisal University, Al-Hasa, SAU

**Keywords:** backache, pandemic, chronic pain, low back pain, covid-19

## Abstract

Background

Low back pain (LBP) is a common musculoskeletal condition that affects individuals worldwide, causing difficulties in daily tasks and social interactions. It can be categorized based on chronicity, with acute, subacute, and chronic forms. The causes of backache vary among patients and can include inflammatory conditions, radiculopathy, pregnancy, trauma, osteoporosis, nerve root compression, cancer, plexopathy, infection, and other spinal diseases.

Aim

The aim is to investigate the association between COVID-19 infection and LBP between all Saudi adults and foreign adults who had positive COVID-19 tests in the eastern region of Saudi Arabia.

Methods

A cross-sectional study was conducted in the Eastern Province of Saudi Arabia over the period from March 2023 to August 2023. Participants were selected by using a convenience sampling method, a sample (n=500) of individuals. The structured questionnaire was used to gather information on sociodemographic variables and COVID-related features. All the statistical calculations were performed using the SPSS software (by IBM) version 29.0.0.

Results

482 participants completed the questionnaire. Out of 482 participants, the majority were females with a number of 372 (77.2%) aged between 20 and 29 years (38.4%). Out of the remaining participants, 110 (22.8%) were males. Most of the participants with a number of 301 (62.4%) were from the Hasa province. This was followed by Qatif (79, 16.4%), Dammam (56, 11.6%), Jubail (25, 5.2%), and others (21, 4.4%). The study revealed that 10.1% of participants reported experiencing back pain. The duration of backaches varied among respondents, with 122 (25.3%) experiencing them from a day to a week, 28 (5.8%) enduring them for six weeks, and 65 (13.5%) reporting a duration of six to 12 weeks. The majority, comprising 267 (55.4%) respondents, were uncertain about the period of their backaches. The prevalence of COVID-19 infection among the participants was 357 (74.1%), and 477 (99.0%) had been vaccinated against COVID-19. Approximately 44.4% of the participants experienced back pain, and out of those, 28.2% reported having pain during quarantine. Among the individuals with back pain, 24.7% attributed it to COVID-19.

Conclusion

This study highlights the significant correlation between back pain and COVID-19, even after the resolution of other symptoms. It underscores the importance of further research into the long-term effects and mechanisms of this association. The findings emphasize the need for healthcare professionals to consider back pain as a potential aspect of the post-COVID-19 symptom profile, ensuring comprehensive care for affected individuals.

## Introduction

The World Health Organization (WHO) reported a global coronavirus pandemic (COVID-19) in 2019, caused by a virus that primarily affects the respiratory system and spreads rapidly through close contact [1]. Fever, cough, and shortness of breath are the three most prevalent complaints associated with COVID-19, albeit the clinical manifestation varies from person to person [2].

In July 2020, there were 14 million active cases [3]. In Saudi Arabia, the rate of spread accelerated; there were 392 cases overall in 2020, and 549518 cases were reported in 2021 [4].

Low back pain (LBP) manifests within the anatomical region spanning from the costal margins to the inferior gluteal folds. It has the potential to present alongside or in isolation from lower extremity pain, posture abnormalities, stiffness, and other related symptoms [5]. There are many types of back pain based on chronicity, LBP can be categorized as acute (six weeks), subacute (six to 12 weeks), and chronic (>12 weeks) [6].

The etiology of back pain entails a diverse range of conditions that can be classified into inflammatory conditions like ankylosing spondylitis and sacroiliitis, pregnancy, trauma, osteoporosis, cancer, plexopathy, and infections [7]. 

Individuals experiencing LBP often report limitations in performing activities of daily living, with difficulties in social interactions and community life being particularly significant for people with LBP [8]. The incidence varies in Saudi Arabia, falling between 53.2% and 79.17% and is frequently reported among medical students [9].

The number of COVID-19-positive patients presenting with back pain was remarkable and the study conducted in Turkey involving 210 patients indicated that 33.1% reported experiencing LBP, revealing a significant correlation between back pain and COVID-19 [10].

A study in Riyadh on 463 adults revealed increased LPB during quarantine from 38.8% to 43.8%. It aimed to explore the COVID-19 LBP association among Saudi and foreign adults in Saudi Arabia's eastern region [11].

## Materials and methods

Methods

A cross-sectional study was conducted from March to August 2023 to investigate the association between COVID-19 infection and LBP between all Saudi adults and foreign adults who had positive COVID-19 tests in the eastern region of Saudi Arabia. We adopted a convenient sampling method and sent an electronic self-administered close-ended anonymous survey through social media. We received a total of 500 survey responses.

Inclusion criteria

This study included all willing individuals of both genders, aged 18-59, residing in the eastern region of Saudi Arabia.

Exclusion criteria

Participants who did not fill out the whole questionnaire were excluded from the study.

Survey

The survey consisted of two sections. The first section collected demographic information, including age, sex, marital status, occupation, and weight. The second section focused on COVID-19-related variables, such as COVID-19 vaccination status, symptoms experienced during the infection, onset of back pain, severity of back pain on a scale of 0 to 10, and duration of back pain.

Data entry and analysis

Data entry was performed by using Microsoft Excel (Microsoft Corporation, Redmond, USA). Statistical Package for the Social Sciences (SPSS) version 21 (IBM Corp., Armonk, NY) was used for statistical analysis. Frequency and percentages were used to describe categorical variables, whereas mean and standard deviation were used to describe the continuous variables. Data comparison was made using the Chi-square test, Fisher's exact test, and independent t-test. A p-value <0.05 was considered statistically significant.

Ethical approval

All data remains strictly confidential, was only utilized exclusively for scientific purposes, and is compliant with the moral standards for studies involving human participants. The dean of scientific research at King Faisal University provided ethical approval for this study to be conducted (KFU-REC-2023-APR-ETHICS781).

## Results

Out of 500 participants initially included, 18 were excluded for not being from the Eastern province, resulting in 482 responses included in the analysis after data cleaning.

Table 1 reveals that out of 482 participants, the majority were females (77.2%), primarily aged 20-29 years (38.4%), and weighed between 40 and 60 kg (50.6%). Most were single (54.1%), with varied occupations including students (39.8%), teachers (11.0%), nurses (13.5%), and pharmacists (10.0%). The sample represented the eastern region, predominantly from Al-Hasa province (62.4%).

**Table 1:** Sociodemographic and other features of patients with back pain (n=482)

		Frequency	Percent
Gender	Females	372	77.2
	Males	110	22.8
Age	18-20 years	117	24.3
	20-29 years	185	38.4
	30-39 years	85	17.6
	40-49 years	76	15.8
	50-59 years	19	3.9
Weight	40-60 kg	244	50.6
	61-80 kg	171	35.5
	81-100 kg	53	11.0
	>100 kg	14	2.9
Marital status	Single	261	54.1
	Married	221	45.9
Occupation	Student	192	39.8
	Teachers	53	11.0
	Nurses	65	13.5
	Pharmacist	48	10.0
	Other jobs	22	4.6
	Unemployed	102	21.2
Region	Eastern	482	100.0
Provinces	Al-Hasa	301	62.4
	Qatif	79	16.4
	Dammam	56	11.6
	Jubail	25	5.2
	Others	21	4.4

**Figure 1:**
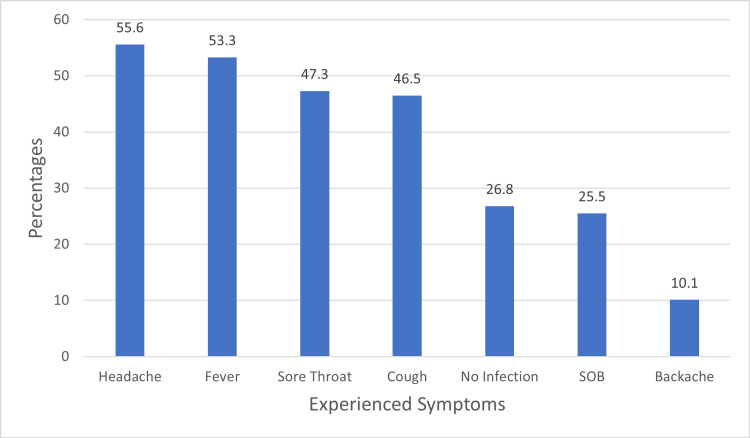
Symptoms experienced by patients with back pain

Figure 1 shows the prevalence of experienced symptoms among respondents. Headache was reported by 55.6%, fever by 53.3%, sore throat by 47.3%, and cough by 46.5%. A smaller portion had no infection (26.8%) or experienced shortness of breath (25.5%), while backache was reported by 10.1% of patients

Table 2 shows the prevalence of COVID-19 and back pain-related features among 482 patients. The majority were infected with COVID-19 (74.1%) and had received the COVID-19 vaccine (99.0%). Around 44.4% experienced back pain, with 28.2% reporting pain during quarantine. 24.7% attributed their back pain to COVID-19. Pain onset varied, with 18.7% experiencing it before the pandemic and 26.8% during COVID-19 infection. Different pain durations were reported, including day-to-week (25.3%), six to 12 weeks (13.5%), and uncertain duration (55.4%). Pain intensity ranged from light (7.9%) to very strong (3.1%).

**Table 2:** Prevalence of COVID-19 and back pain-related features in patients (n=482)

		Frequency (n=482)	Percent
Infected with COVID-19	Yes	357	74.1
Received COVID-19 vaccine	Yes	477	99.0
Ever experienced back pain	Yes	214	44.4
Suffered from back pain in quarantine	Yes	136	28.2
You think back pain due to COVID-19	Yes	119	24.7
When did pain begin	Before COVID-19 pandemic	90	18.7
	During COVID-19 infection	129	26.8
How long the back pain lasted	Day to week	122	25.3
	For 6 weeks	28	5.8
	6 to 12 weeks	65	13.5
	Don’t know	267	55.4
Pain scale assessing intensity of pain	1-3 (light)	38	7.9
	10 (very strong)	15	3.1
	4-6 (medium)	122	25.3
	7-9 (strong)	56	11.6

**Figure 2:**
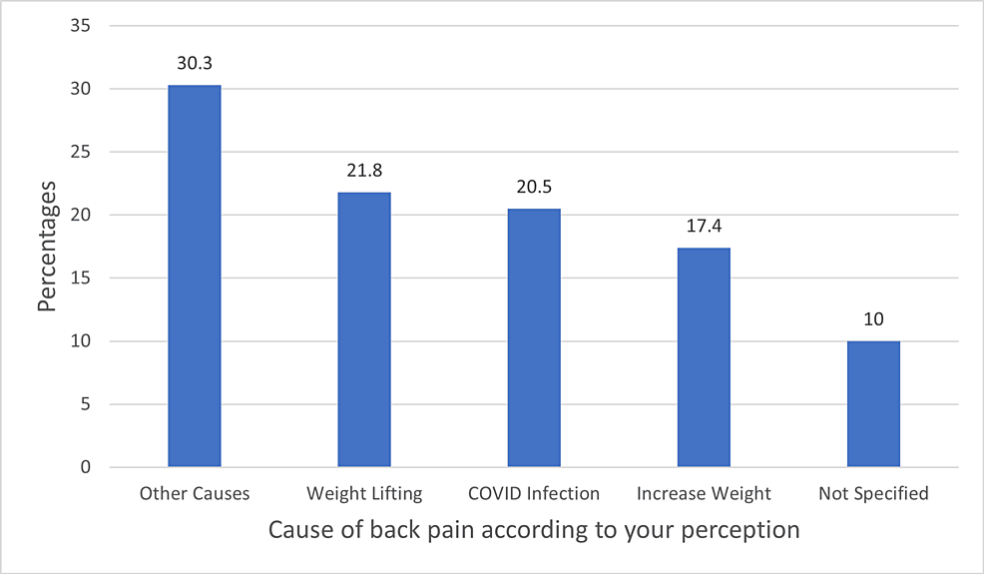
Patient perceptions about the reason for backache

Figure 2 shows the perceived causes of back pain. Among the respondents, 30.3% attributed it to other causes, 21.8% to weight lifting, 20.5% to COVID-19 infection, 17.4% to increased weight, and 10% stated no specific cause.

Table 3 shows a significant association between back pain and various COVID-19-related features and sociodemographic factors. Notably, COVID-19 infection is linked to back pain (p<0.001), with a perception of COVID-19-related pain (p<0.001) and experiencing pain during quarantine (p<0.001) strengthening this association. Pain onset during COVID-19 infection also holds significance (p<0.001). Among sociodemographic features, age (p=0.004), occupation (p=0.016), marital status (p=0.002), and weight (p=0.035) show noteworthy correlations with back pain prevalence.

**Table 3:** Association of back pain with COVID-19-related features and sociodemographic features (n=482)

		Pain in your back		Sig. value
		No	Yes	
COVID-19-related features				
Infected with COVID-19 virus	No	90	35	<0.001
	Yes	178	179	
According to your opinion, back pain is due to COVID-19 infection	No	252	111	<0.001
	Yes	16	103	
Suffer from back pain in quarantine	No	230	116	<0.001
	Yes	38	98	
When pain start	Before COVID-19 pandemic	3	87	<0.001
	During COVID-19 infection	10	119	
Ever received COVID-19 vaccine	No	5	0	0.069
	Yes	263	214	
Sociodemographic features				
Gender	Female	205	167	0.688
	Male	63	47	
Age	18-20 years	78	39	0.004
	20-29 years	108	77	
	30-39 years	39	46	
	40-49 years	32	44	
	50-59 years	11	8	
Occupation	Student	124	68	0.016
	Teachers	21	32	
	Nurses	36	29	
	Pharmacist	26	22	
	Other jobs	10	12	
	Unemployed	51	51	
Provinces	Al-Hasa	165	136	0.575
	Qatif	41	38	
	Dammam	33	23	
	Jubail	14	11	
	Others	15	6	
Marital status	Single	162	99	0.002
	Married	106	115	
Weight	40-60 kg	151	93	0.035
	61-80 kg	85	86	
	81-100 kg	24	29	
	>100 kg	8	6	

## Discussion

Certainly, while there is an association between back pain and COVID-19, it is important to note that not all back pain is directly caused by the viral infection. However, research has indicated a relationship between back pain and COVID-19. It is worth noting that body aches and pains, including back pain, are common symptoms of COVID-19 and can persist even after other symptoms have subsided [12]. However, it is also crucial to consider that back pain can have various causes, and not every instance of back pain is linked to COVID-19.

The current study in the Eastern Province of Saudi Arabia found that a significant portion of COVID-19-infected individuals experienced back pain, with a quarter attributing it to the infection, and approximately one-third reporting medium to strong intensity pain, aligning with research indicating a correlation between COVID-19 and musculoskeletal symptoms including back pain [13].

Recently, studies have shown that back pain can be a symptom of COVID-19. In a 2022 study of hospitalized people with COVID-19, 69.3% reported pain, with 43.6% reporting back pain and 33.1% reporting lower back pain. Additionally, body aches and pains, including back pain, are common symptoms of COVID-19 and can persist long after other symptoms subside [14]. Furthermore, another study from 2020 reported that 69.3% of people with COVID-19 experienced pain as a symptom, with back pain being one of the most frequently reported types of pain [15]. Markedly, joint pain is documented as the third most frequent (27.3%) following fatigue (53.1%) and dyspnea (43.4%) [16]. Moreover, anxiety for chronic pain syndromes after COVID-19 started among the rehabilitation and pain control communities [17,18]. In Saudi Arabia, Abumunaser et al. reported an increase in the prevalence of LBP, with only 44.8% of participants presenting with LBP before quarantine and 59.4% having it during quarantine [19]. Another study in Riyadh revealed that LBP prevalence before the quarantine was 38.8%, which increased to 43.8% after the quarantine. The LBP intensity pointedly increased during the quarantine. The lower back was also the most reported musculoskeletal pain area [11]. In Bangladesh, Ali M et al. found that Overall, 20% of participants reported LBP; however, the prevalence of LBP was significantly higher among patients with post-acute COVID-19 compared with their counterparts (24.4% vs 15.7%, P=0.001) [20]. On the other hand, Caputo EL et al. in Brazil revealed that the prevalence of LBP did not change significantly from before (74.7%) to the first months of the pandemic (74.2%) but increased pain levels and a higher likelihood for activity limitation due to LBP were observed [21].

It is important to note that back pain can have many causes, and not all back pain is related to COVID-19. Experiencing back pain, in addition to any symptoms related to COVID-19, people should contact their healthcare provider [22].^ ^Additionally, COVID-19 is now recognized as a multiorgan disease, with a wide range of clinical issues [23,24]. Therefore, COVID-19 may induce spinal derangement causing LBP. COVID-19-induced spinal muscle weakness and facet joint pain may stand behind experiencing LBP. Other factors of LBP, including depression, anxiety, or cognitive dysfunction, which COVID-19 induced by COVID-19 may also trigger LBP among patients with post-acute COVID-19 [25,26].

One limitation of this study is the use of convenience sampling, which may introduce selection bias. This could affect the generalizability of the findings to other regions or populations within Saudi Arabia or globally. Another limitation is the reliance on self-reported data through a structured questionnaire. Self-reported symptoms and attribution of back pain to COVID-19 may be subject to recall bias or misinterpretation by participants. Longitudinal studies or prospective designs would be better suited to investigate the temporal association and potential long-term consequences of COVID-19 on back pain instead of cross-sectional design.

Overall, while this study provides valuable insights into the association between back pain and COVID-19, these limitations should be taken into account when interpreting the results, and further research is needed to validate and expand upon these findings.

## Conclusions

In conclusion, it is imperative to acknowledge that not all instances of back pain can be directly attributed to viral infections. However, existing research highlights a significant association between back pain and COVID-19. This discomfort is commonly reported as a symptom of the infection and may persist even after other symptoms have abated. This emphasizes the necessity for a sustained investigation into the long-term ramifications and potential mechanisms underlying the persistence of back pain in individuals recovering from COVID-19. As our understanding advances, it is crucial for researchers to remain vigilant in exploring the role of back pain in the post-COVID-19 symptom profile, thereby facilitating comprehensive care strategies for affected individuals.
